# Urine metabolomics in neonates with late-onset sepsis in a case-control study

**DOI:** 10.1038/srep45506

**Published:** 2017-04-04

**Authors:** Kosmas Sarafidis, Anastasia Chrysovalantou Chatziioannou, Agathi Thomaidou, Helen Gika, Emmanouel Mikros, Dimitra Benaki, Elisavet Diamanti, Charalampos Agakidis, Nikolaos Raikos, Vasiliki Drossou, Georgios Theodoridis

**Affiliations:** 1 1st Department of Neonatology, School of Medicine, Aristotle University of Thessaloniki, Greece; 2 School of Chemistry, Aristotle University of Thessaloniki, Greece; 3 Laboratory of Forensic Medicine and Toxicology, School of Medicine, Aristotle University of Thessaloniki, Greece; 4 School of Pharmacy, National and Kapodistrian University of Athens, Greece

## Abstract

Although late-onset sepsis (LOS) is a major cause of neonatal morbidity and mortality, biomarkers evaluated in LOS lack high diagnostic accuracy. In this prospective, case-control, pilot study, we aimed to determine the metabolic profile of neonates with LOS. Urine samples were collected at the day of initial LOS evaluation, the 3^rd^ and 10^th^ day, thereafter, from 16 septic neonates (9 confirmed and 7 possible LOS cases) and 16 non-septic ones (controls) at respective time points. Urine metabolic profiles were assessed using non-targeted nuclear magnetic resonance spectroscopy and targeted liquid chromatography-tandem mass spectrometry analysis. Multivariate statistical models with data from either analytical approach showed clear separation between the metabolic profiles of septic neonates (both possible and confirmed) and the controls. Metabolic changes appeared to be related to disease progression. Overall, neonates with confirmed or possible LOS exhibited comparable metabolic profiles indicating similar metabolic alternations upon the onset of clinical manifestations. This methodology therefore enabled the discrimination of neonates with LOS from non-septic individuals, providing potential for further research toward the discovery of LOS-related biomarkers.

Late-onset sepsis (LOS), variably defined for epidemiological purposes as occurring after 72 hours to 6 days of life^1^,^2^,^3^, is a major health care issue, especially for neonates born prematurely. Although antibiotic treatment and supportive care have considerably decreased deaths related to LOS, mortality still remains high, particularly when associated with Gram (−) microbes and/or septic shock^4^,^5^,^6^. Moreover, survivors are at increased risk for adverse neurological^4^,^7^ and other neonatal morbidities such as bronchopulmonary dysplasia^6^,^7^.

Early diagnosis of sepsis and timely, prompt initiation of treatment have been shown to improve survival and functional outcome^8^. Nevertheless, detection of septic neonates is difficult, mainly due to the non-specific clinical signs and the relative diagnostic inaccuracy of the available biomarkers^9^. In a recent study on the management of LOS in five European countries, the expert panel-derived (clinical and laboratory) criteria used were reported to perform reasonably well in identifying patients with culture proven sepsis^5^, but evidently there is a need for better means/techniques to diagnose LOS.

Metabolomics is a bio-analytical approach aiming toward the comprehensive profiling of small molecule content of the examined sample. Urine metabolomics has been increasingly used for the study of biochemistry and biomarker discovery in disease as it can provide important information on the overall metabolic status of an individual. Moreover, urine seems to be more suitable for metabolic analysis in the neonatal population as it offers significant methodological advantages compared to e.g., blood-based sampling; the risk of iatrogenic anemia in critically-ill infants is minimized and issues associated with the need for invasive procedures and relatively large sample volumes in these tiny patients are eliminated^10^.

In this context, metabolomics has also been applied for the assessment of critical illness such as sepsis. To date, metabolomics studies in sepsis have been conducted in animals^11^ as well as in adult^12^,^13^,^14^ and pediatric patients^15^, covering the whole sepsis spectrum from endotoxemia to systemic inflammatory response syndrome (SIRS), and septic shock. Overall, these investigations showed significant promise on disease profiling and biomarker identification in sepsis^16^. However, to date, relevant studies in neonatal sepsis are sparse^17^.

We hypothesized that metabolomics could provide potential biomarkers of neonatal sepsis. Thus, the aim of the present study was to investigate metabolic changes related to LOS, employing two analytical platforms: proton nuclear magnetic resonance (^1^H-NMR) spectroscopy and liquid chromatography-tandem mass spectrometry (LC-MS/MS).

## Results

### Study population

Sixteen septic neonates (9 with confirmed and 7 with possible LOS) and 16 controls were studied. The two groups were comparable regarding demographic-perinatal characteristics (Table 1). One neonate with confirmed and one with possible LOS died; all controls were discharged home. Organisms isolated in blood cultures were mainly gram (−) microbes [*Klebsiella pneumonia* (n = 4), *Klebsiella oxytoca* (n = 1), *Enterobacter cloace* (n = 2)]; *Group B Streptococcus* and *Candida famata* were isolated in two cases. Results of the initial laboratory investigation for LOS are also shown in Table 1. As expected by study design, controls had a negative workup for sepsis (Table 1). Data on the demographic-perinatal characteristics as well as of the initial laboratory investigation for sepsis of the neonates with confirmed and possible sepsis, separately, are also presented in Table 1.

### ^1^H-NMR spectroscopic data

After an extensive search of the ^1^H-NMR spectroscopic profiling-data using Chenomx software, a local spectral library and 2D spectra for identification, 110 peaks were selected and their signals were integrated. This approach enabled the elimination of interfering signals deriving from pharmaceuticals and/or their metabolites.

**Figure 1:**
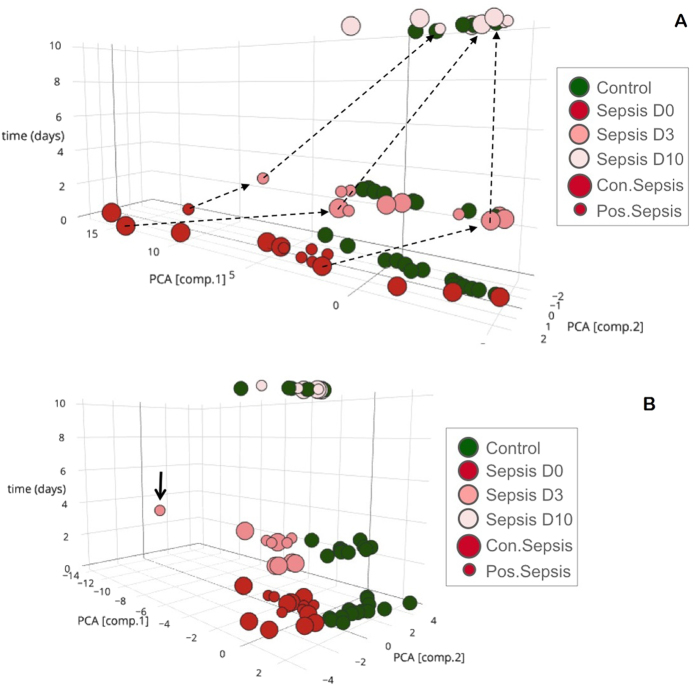
PCA scores plots. First and second component explaining the variation among data are plotted at three time-points D0, D3 and D10. Top Frame: ^*1*^*H*-*NMR data*: Samples of DO show clear separation of the two groups (septic patients versus controls). On D3 separation is not as evident, while on D10 samples cluster all together Bottom frame: *LC*-*MS/MS data*: Samples of DO and D3 show clear separation of the two groups (septic patients versus controls). On D10 samples cluster all together. The arrow indicates a deceased septic neonate on day 3. Green circles correspond to controls and red circles to sepsis subjects (smaller circles denote possible and large circles confirmed sepsis). Pink correspond to day 3 and light pink to day 10.

Analysis of the spectral data by multivariate analysis showed that, even by unsupervised Principal Component Analysis (PCA), septic infants (confirmed and possible) could be discriminated from non-septic ones (controls) at the time point of sepsis suspicion (D0). Figure 1A shows scores plots of the first two components, which describe the highest variation among the samples over the duration of the investigation. PCA models were generated separately for each time point, and in the plot the corresponding score plots are presented on a vertical time axis, to indicate the “trajectory” of the disease progression as indicated by the changes in urine composition. It can be seen that D0 samples from septic neonates (confirmed and possible sepsis) are co-located in the PCA plot and separately from their paired controls. This trend was also observed on D3 although differentiation was less clear, whereas on D10, when the LOS infants no longer show symptoms, samples from both patients and controls show no difference. To determine which metabolites were responsible for group differentiation, a number of visualization plots and evaluation tools, both multivariate and univariate, were applied (as described in the experimental section). Table 2 shows log2fold changes of the ^1^H NMR signals, and the p-values of T-tests for significant metabolites/peaks. In total 10 peaks were found to pass all evaluation filters for D0 and these are given in Table 2 where septic cases subgroups (confirmed and possible) are compared independently to the control group. If the two septic infant sub-groups are considered as one group (disease group) a number of additional metabolites were highlighted by both multivariate and univariate statistical analysis: acetone, sarcosine, leucine and dimethylamine exhibit log2fold change higher than 1 (twofold signal intensity) and p-values < 0.05. Results from ^1^H NMR spectroscopy for D3 and D10 showed no significant statistical alteration to these metabolites. Supplementary Fig. S1 provides the ROC values and box plots (median values) for the most statistically significant metabolites (D0).

### LC-MS/MS data

Differentiation of septic (both confirmed and possible) from control patients was revealed by PCA analysis. PCA scores plots (Fig. 1B) show patient and control groups clearly separated on D0 and D3 in t1 vs. t2 space. However, this was not the case on D10. Of note, a case of a deceased septic infant appeared as clear outlier on D3 (no sample could be collected on D10). As with the ^1^NMR spectral data, cases of possible sepsis (D0) were found to cluster together with of those with confirmed sepsis (D0) in the PCA plots. It should be stressed that in general, cases of confirmed and possible sepsis could not be discriminated by multivariate analysis. Even when models were built only for these two classes of samples, PCA did not show any separation, while no valid Partial Least Square-Discriminant Analysis (PLS-DA) and Orthogonal Partial Least Square-Discriminant Analysis (OPLS-DA) models could be built.

Subsequently, OPLS-DA models were built to allow the identification of metabolites contributing to group differentiation (D0). An OPLS-DA scores plot showing the two separate classes of samples is illustrated in Supplementary Fig. S2. Permutation tests, CV-ANOVA p-value (p ≪ 0.001) and Q2Y value 0.75 indicated the model’s good predictability (permutation plot shown in Fig. S2). Loadings plot (Supplementary Fig. S3), depicts the contribution of metabolites to group’s classification.

OPLS-DA models of D3 urine (validity proven by p < 0.001, Q2Y 0.77) again showed clear differentiation between septic neonates and controls. Supplementary Fig. S4 depicts the OPLS-DA scores plot for the two groups at D3, along with the permutation plot. However OPLS-DA models built on samples from D10, were not able to classify the two groups (permutation plot invalid and CV-ANOVA p > 0.05), indicating that the metabolic profiles of patients and controls no longer differed in terms of metabolic phenotypes.

With regard to the contribution of specific metabolites to group classification, in D0 samples were found to significantly differ in the signals of 17 metabolites measured by LC-MS between septic neonates and controls. Among the significant metabolites, three were also found significant in the ^1^H NMR spectral data and are noted with asterisk in Table 3. Three metabolites were still significantly different on D3 passing all evaluation criteria while none was different on D10. When all diseased infants were considered as one group, seven additional metabolites were found to continue to exhibit significant alteration also on D3: valine, phenylalanine, taurine, fumaric acid, lactic acid, glucose and riboflavin, which all showed log2fold change >1.6.

ROC curves/values and box plots for significant metabolites are given in Supplementary Fig. S5.

## Discussion

In this study, the potential of metabolomics to identify biomarkers related to LOS in the urine of neonates was investigated. The results show that the metabolic profile of neonates with proven and possible LOS was markedly different compared to those without sepsis. The metabolic alternations seen were mainly relevant to energy producing biosynthetic pathways and basic structural components of the organism, contributing significantly to the clear discrimination of septic neonates using metabolomic analysis. Neonates with confirmed and possible sepsis exhibited similar metabolic profiles under the applied methods, thus, indicating similar underlying biological derangements. This fact lends further support to the notion that septic neonates, and those fulfilling the accepted criteria for LOS, but not confirmed by culture should be treated in the same manner, irrespective of the blood culture results.

The triage of septic neonates from those with other severe conditions is a major challenge in everyday clinical practice. In this investigation, initially using ^1^H-NMR spectroscopy for the analysis of the urine samples in an attempt to obtain an overall view of the metabolic profile, we were able to document a clear separation between neonates fulfilling the diagnostic criteria for LOS and non-septic controls. Non-targeted metabolomics investigation revealed significant changes in 10 urine metabolites. With the targeted metabolomics approach (LC-MS/MS) that was applied next for the quantification of additional important metabolites, the selection of which was based on the existing literature, we found statistically significant alternation in 17 metabolites in the urine, three of which were also found to be statistically significant by ^1^H-NMR spectroscopy.

In the present study, 7 (43.8%) out of the 16 neonates evaluated had possible (blood culture negative) LOS, whereas the disease was strongly indicated by the clinical signs as well as laboratory indicators of sepsis. This subgroup of neonates could be clearly distinguished from controls using metabolomics. Interestingly, however, no discrimination could be made between neonates with possible and confirmed LOS on the clinical onset of the disease and the third day thereafter. Nevertheless, the finding of common metabolites in the neonates with confirmed and possible LOS on day 0 is suggestive of similar underlying pathophysiological mechanisms, regardless of the blood culture positivity. Positive blood culture is the gold standard for the diagnosis of sepsis, but novel diagnostic methods demonstrate a suboptimal diagnostic accuracy of blood cultures for the detection of LOS^18^. The presence of sepsis manifestations with negative blood cultures is a real and common observation in adults^19^,^20^ and children^21^ including neonates^5^,^22^. As many as 60% of the neonates enrolled in the International Neonatal Immunotherapy Study, in which the effect of intravenous immunoglobulin was evaluated for the treatment of neonatal sepsis, were reported to have blood culture-negative sepsis^23^. In any case, our findings support the clinical decision to administer antibiotics in neonates with clinical and laboratory evidence of sepsis despite negative blood cultures. It is worth noting that, no discrimination could be made on the day 10 of sepsis, when metabolic excursions from normal had recovered in the affected infants along with the clinical and laboratory improvement of sepsis. Characteristically, septic infants shifted towards controls on unsupervised PCA analysis based on both ^1^H-NMR spectroscopy and LC-MS/MS (Fig. 1). In a previous study, nine infected neonates with early- and late-onset sepsis were distinguished from 16 controls after urine analysis by ^1^H-NMR and GC-MS. However, these were only evaluated at one time-point, while no clarification was made as to whether they suffered clinical or blood culture proven sepsis^17^. On the basis of our results, urine metabolomics show potential as a diagnostic means, not only for the reliable detection of septic neonates, but also for monitoring response to therapy.

An interesting hypothesis in biomarker research including sepsis is the potential of predicting disease development considerably sooner, even days before the appearance of clinical signs. Circulating sepsis indicators have been reported to increase in septic neonates up to 2 days before clinical suspicion^24^ while the earlier identification of very low birth weight infants at risk for imminent LOS after continuous monitoring of heart rate characteristics (decreased variability and transient decelerations partly related to inflammatory response to sepsis), led to a significant reduction in sepsis-associated mortality from 19.6% to 11.8%^25^. Indeed, screening based on metabolic or proteomic changes could enable studies towards earlier diagnosis, and this attractive approach should clearly be evaluated in future studies using the appropriate design for sampling.

**Figure 2:**
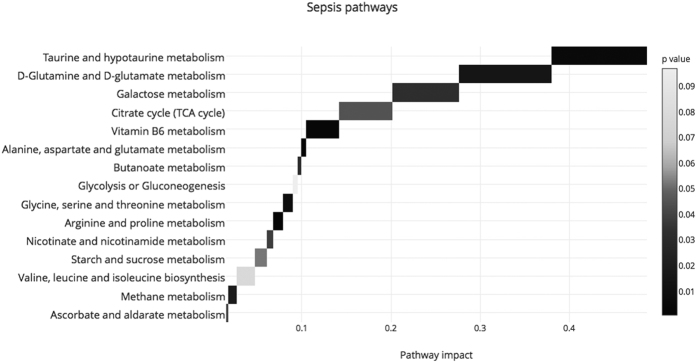
Higher impact pathways involving significant metabolites of both techniques as resulted from the online web software MetaboAnalyst. The significance of a model increases in the y-axis. The color bar indicates the p-value of the pathway; darker color corresponds to more significant p-value (smaller value).

Our data show (Fig. 2) that several metabolic pathways are influenced in neonatal sepsis^26^. In particular, we noted significant elevations in the amounts of urinary taurine and hypotaurine, the presence of which had a significant statistical impact on the differentiation of septic neonates from those without the disease. As taurine has anti-inflammatory and anti-oxidant properties^27^, increased concentrations of the specific amino acid may have a cytoprotective effect. Similarly to our results, Su *et al*. reported significantly higher taurine concentrations in adult patients with SIRS and sepsis^28^.

Glutamine and glutamate metabolism also seems to play a crucial role during sepsis in neonates. Critically-ill adult patients characteristically have depleted glutamine, presumably due to increased metabolic demands^14^. Glutamine is important for several organ functions including gut integrity (fuel for enterocytes), immunologic response (proliferation and function of immune cells) and antioxidative balance (glutathione synthesis)^29^. Deamidation of glutamine produces glutamate, a precursor of the neurotransmission inhibitor gamma-aminobutyric acid^30^. In preterm infants glutamine supplementation has been suggested as beneficial. However, recent Cochrane meta-analysis did not find an effect of this strategy on mortality (or major neonatal morbidities including the incidence of invasive infection or necrotizing enterocolitis)^31^.

Altered glucose metabolism is a major metabolic disturbance in septic neonates manifesting hypo- or hyperglycemia. In this study, the urinary glucose concentration was higher in septic neonates compared to controls, although this was significantly increased only with metabolomics. We also documented significantly increased amounts of pyruvate and lactate in the urine of sepsis cases possibly representing sepsis-associated hypoperfusion and/or hypoxia favoring lactate production or reduced lactate clearance^32^. Lactic acidosis and increased glucose requirements are known early metabolic responses in septic preterm infants^33^. Moreover, previous studies in adults documented the significance of elevated serum lactate as predictors of in-hospital mortality in infected-septic patients^34^. Alternatively, elevated lactate may represent decreased mitochondrial oxidative metabolism (oxidative phosphorylation) described in critically-ill patients^35^. Defective operation of the citric acid (Krebs) cycle in sepsis^36^ could also explain significantly lower concentrations of two of its intermediates alpha-ketoglutaric acid and fumarate in our group of septic infants compared to controls.

Inosine, a product of the purine degradation pathway was found to be present in increased amounts in samples from septic neonates in the present study, while hypoxanthine was also increased but marginally failed the evaluation criteria. Accumulation of purines is believed to result from the cellular destruction following sepsis and the degradation of high-energy phosphate compounds. Xanthine oxidase activation is a leading factor for the oxidant stress and a marker of poor prognosis in patients with sepsis^37^,^38^.

We found several amino acids to be significantly increased in samples from septic vs. non-septic neonates. Previous studies in adults with sepsis have also documented altered plasma amino acids concnetrations^28^ implying a disturbed protein metabolism^26^,^28^ associated with increased mortality^28^,^39^. In adults, skeletal muscle protein wasting is a prominent feature of the metabolic response to sepsis^40^.

Additionally, we noted decreased quantities of trimethylamine N-oxide (TMAO) in urine from septic neonates. Mickiewicz *et al*., also reported on TMAO in the serum of adult patients with septic shock^41^, but, in contrast to our data, specific concentrations were reported to be significantly increased compared to controls. As TMAO is an intestinal microbiota-generated amine derived from phosphatidylcholine^42^, alternations of the gut bacteria in neonates consequently to sepsis *per se*^43^ or antibiotic treatment^44^ could affect its production. In adults, plasma TMAO concentrations are markedly suppressed after the administration of antibiotics^42^.

Finally, in our study, septic neonates had significantly lower amounts of certain vitamins of the B complex such as riboflavin (vitamin B2) and nicotinamide (vitamin B3) as compared to non-septic ones. These vitamins are coenzymes or precursors of cofactors involved in several important biochemical pathways (e.g., the Krebs cycle), and sepsis predisposition to deficiency in several micronutrients^45^ could provide an explanation for their reduced levels.

Beyond the combined application of two analytical platforms, the present study also has other strengths. As far as we know, this is the largest metabolomic study to have been performed in septic neonates, where subjects were serially evaluated at three time-points during the acute phase and upon recovery from the disease. Moreover, we evaluated urine samples, with the aforementioned methodological advantages of the specific biological fluid in neonates. However, an obvious limitation is that it represents a pilot study conducted in a single center, thus limiting the number of the septic neonates that could have been enrolled. Although the sample size was adequate to reliably document differences in the metabolic profile between septic neonates, irrespective proven or suspected LOS status, and controls, the number of the enrolled subjects did not allow the evaluation of other important variables. For example, Gram (+) and Gram (−) bacteria may have elicited different metabolic responses, but we failed to prove this as the majority of cases suffered from Gram (−) sepsis. Similarly, we could not construct models based on mortality due to the very small number of cases (only two infants) with this unfavorable outcome. Interestingly, one could expect an altered metabolic profile related to neonatal maturation. Previous investigations have clearly documented developmental differences in inflammatory response to sepsis^46^ but in the present study using multivariate analysis gestational age was found not to be a significant independent factor associated with the metabolic profile. This was also the case with some external factors that were expected to differ in septic and non-septic neonates such as the feeding mode and drug intake (inotropes, antibiotics) and are known to affect urine composition^9^. It is worth noting that gestational and post-natal age at the 1^st^ urine sampling was specifically taken into consideration at study design because of their possible influence on urine metabolic profiles and, therefore, cases were matched to controls for these factors to the best possible level. Nevertheless, the association between risk factors and outcome (in this case of the clinical characteristics with metabolic profiles) is difficult to evaluate in case-control studies particularly when the patient number is relatively small. Moreover, over-matching can make it difficult to find enough controls in the clinical setting. For all the above reasons, the validation of our results in the context of larger multi-center studies would be justified, especially in relation to important clinical outcomes such as those aforementioned.

In conclusion, neonates with confirmed and possible sepsis at the onset of clinical manifestations showed a different metabolic profile compared to those without sepsis allowing their clear discrimination with the use of ^1^H-NMR and LC-MS/MS-based urine analysis. Results of this pilot study support the usefulness of metabolomics in exploring underlying biochemical mechanisms of the disease, that could -after validation- provide novel candidate mechanisms to confirm or follow-up the progression of neonatal LOS.

## Materials and Methods

### Ethical approval

The study protocol was approved by the School of Medicine ethics committee of the Aristotle University of Thessaloniki. An informed written consent was obtained from all parents/guardians before enrolling their child in the study. All experiments were performed in accordance with relevant guidelines and regulations.

### Study population

This prospective, case-control, pilot study was performed in a single, tertiary-level neonatal intensive care unit during a two-year period (September 2013-August 2015). Eligible for the study were preterm and term neonates who needed evaluation for LOS (sepsis presenting after the first 72 hours of life). Cases included neonates with confirmed (positive blood cultures for microbes or fungi) or possible LOS (clinical and laboratory evidence of sepsis but negative blood cultures). For LOS diagnosis, we adopted the criteria defined in 2010 by an Expert Meeting of the European Medicines Agency on Neonatal and Paediatric Sepsis^3^; these criteria required the presence of at least two clinical and two laboratory parameters and have already been evaluated in neonates with LOS^5^. Immediately after the sepsis workup was performed, neonates received sepsis treatment according to the protocols applied in our department.

In this study, we compared septic neonates with a group of non-septic ones (controls) receiving intensive care who were stable, less-severely ill and, also, we considered the probable influence of gestational and post-natal age on urine metabolic profile. To this end, each septic neonate was matched to one control of the same gestational age (±one week) and day of life (±3 days but always above 72 hours of life) at the 1^st^ urine sampling. Controls were identified from hospitalized neonates who had no medical problems or were recovering from minor ones (uncomplicated preterm infants, mild respiratory distress, jaundice, etc.) but required sepsis screening, thereafter, for an event such as apnea or mottled skin, proved to have no laboratory evidence of sepsis and, thus, judged as not fulfilling sepsis criteria. The latter infants received either no antibiotics or were given a short course (3–4 days) during the time period in which blood culture results were pending according to our center protocol.

Neonates born to mothers with clinical chorioamnionitis or those who had early-onset sepsis (≤day 3 of life), known congenital infections and anomalies, medium/severe perinatal asphyxia or inborn errors of metabolism were excluded a priori from enrollment. Prior LOS episode, refusal of parental consent, inadequate selection of urine samples, and physical absence of the researcher were also exclusion criteria. Demographic, perinatal and clinical-laboratory data related to LOS were recorded in all neonates.

### Sampling

Urine samples were collected from cases and controls at the day of the initial evaluation for LOS and enrollment in the study, respectively, representing day 0 (D0) and on the 3^rd^ (D3) and 10^th^ day (D10), thereafter, using plastic bags or through a bladder catheter placed for clinical reasons. Samples were centrifuged and supernatants were stored at −80 °C until analysis. Further details on sample processing are provided in Supplementary Material.

### Analysis

^1^H-NMR spectroscopy was performed on an Avance III 600 MHz spectrometer (Bruker BioSpin GmbH) using standard 1D NOESY pulse sequence (noesypr1d). For each sample, 128 scans were collected in 64-k data points over a spectral width of 14 ppm. MNova NMR was used for peak integration purposes and Chenomx software for signal identifications. Subsequently samples were analysed by LC-MS/MS based on an in-house method described previously^47^. The targeted method focused on 108 key hydrophilic metabolites involved in central metabolic pathways and provides complimentary data and strong quantification potential. Further details on the methods are given in Supplementary Information.

### Statistics

The MetSizeR R-package was used for the calculation of the sample size needed to find significant changes in 10–30% out of a total of 90–110 metabolites studied. The target false discovery rate (FDR) was set at 0.05. A sample size of 15 subjects per group was found adequate for the goals of our study.

MedCalc software (Version 11.1.1, MedCalc Software, Belgium) was used for statistical analysis of patient characteristics. Continuous variables were expressed as mean ± SD. The t-test was used for comparisons between two study groups and the Fisher’s exact test was used for the categorical variables.

Multivariate statistical analysis (PCA, OPLS) was performed using SIMCA 13 (Umetrics, Umea, Sweden) with UV scaling. Coefficient plots, VIP plots, loading plots, Receiver Operating Characteristic (ROC) curves and univariate statistics (t-test) were used to find the significant metabolites differentiating the groups. Differences were considered statistically significant when the p value was <0.05.

Associated biochemical pathways were investigated using MetaboAnalyst 3.0^48^.

## Additional Information

**How to cite this article:** Sarafidis, K. *et al*. Urine metabolomics in neonates with late-onset sepsis in a case-control study. *Sci. Rep.* **7**, 45506; doi: 10.1038/srep45506 (2017).

**Publisher's note:** Springer Nature remains neutral with regard to jurisdictional claims in published maps and institutional affiliations.

## Supplementary Material

Supplementary Information

## Figures and Tables

**Table 1:** Demographic, perinatal and clinical-laboratory characteristics.

Descriptive variable	Septic neonates			Controls	p-value*
	All cases (n = 16)	Confirmed sepsis (n = 9)	Possible sepsis (n = 7)	(n = 16)	
**Demographics-perinatal characteristics**					
Gestational age (weeks, mean ± sd)	34.7 ± 3.6	36 ± 3.1	33 ± 3.7	35 ± 3.2	0.796
Birth weight (g, mean ± sd)	2427 ± 935	2654 ± 987	2134 ± 840	2438 ± 880	0.974
Male sex (n, %)	7 (43.7)	4 (44.4)	3 (42.8)	4 (25)	0.457
Preterm (n, %)	11 (68.8)	5 (55.5)	6 (85.7)	11 (68.8)	1
Apgar score1 min (mean ± sd)5 min (mean ± sd)	7.2 ± 1.18.4 ± 0.6	7.2 ± 1.38.3 ± 0.7	7.3 ± 0.78.5 ± 0.5	7.7 ± 0.68.9 ± 0.5	0.1280.048
Caesarian section (n, %)	11 (68.8)	7 (77.7)	4 (57.1)	9 (56.2)	0.715
Prenatal steroid (n, %)	9 (56.2)	3 (33.3)	6 (85.7)	8 (50)	0.724
Small for gestational age (n, %)	1 (6.2)	1 (11.1)	0 (0)	1 (6.2)	1
Premature rupture of membranes >18 hrs (n, %)	1 (6.2)	0 (0)	1 (14.2)	1 (6.2)	1
Time of 1st urine sampling (day, mean ± sd)	9.7 ± 10.8	5.6 ± 1.3	15.1 ± 15.3	13.1 ± 10.1	0.394
**Clinical (day 0)**					
Invasive mechanical ventilation (n, %)	11 (68.7)	6 (66.6)	5 (71.4)	0 (0)	<0.001
Inotropes (n, %)	8 (50)	4 (44.4)	4 (57.1)	0 (0)	0.002
Antibiotics (n, %)	16 (100)	9 (100)	7 (100)	5 (31.2)	<0.001
Full enteral feeding (n, %)	6 (37.5)	4 (44.4)	2 (28.6)	16 (100)	<0.001
**Laboratory findings**					
C-reactive protein (mg/dL)Day 0Day 1	43.6 ± 34.499.5 ± 56.9	48.8 ± 43.3106 ± 63.1	36.7 ± 18.888.3 ± 49.7	All <3.19-—	Not applicableNot applicable
White blood count (K/μL, mean ± sd) - day 0	13.7 ± 10.3	13.5 ± 11.6	14.1 ± 9.2	10.6 ± 2.9	0.279
Immature to total neutrophil ratio (>0.2) - day 0	6 (37.5)	3 (33.3)	3 (48.9)	0 (0)	0.017
Platelet count (K/μL) - day 0	132 ± 98	106 ± 82	165 ± 114	374 ± 163	<0.001
Blood lactate (mg/dL) - day 0	19.3 ± 6.7	21.7 ± 7.8	16.1 ± 3.3	13 ± 4.2	0.007
Blood glucose (mg/dL) - day 0	112 ± 44	115.5 ± 50	108 ± 38	91 ± 12	0.091
Serum creatinine (mg/dL)Day 0Day 3	0.68 ± 0.260.66 ± 0.19	0.65 ± 0.250.66 ± 0.23	0.72 ± 0.290.67 ± 0.16	0.57 ± 0.140.63 ± 0.1	0.2260.716

*p-value refers to comparison between all cases of septic neonates and controls (n = 16).

**Table 2:** Metabolites found by ^1^H-NMR to have significantly changed in septic neonates at the onset of the disease (D0).

metabolites	ppm	D0 possible		D0 confirmed	
		p value	log2fold change	p value	log2fold change
Maltose	5.42	p < 0.05	0.83	p < 0.05	1.48
Glucose	3.28	p < 0.05	1.46	p < 0.05	2.21
Biotin	2.78	p < 0.05	1.16	p < 10^−2^	2.28
Methylamine	2.60	p < 0.05	0.89	p < 10^−2^	1.77
Unknown	2.78	p < 0.05	0.78	p < 10^−2^	1.76
Inosine	8.35	p < 10^−2^	0.78	p < 0.05	1.27
Methylguanidine	2.83	p < 0.05	0.70	p < 0.05	1.11
Creatine	3.00	p < 0.05	1.95	p < 0.05	3.26
Myo-inositol	3.55	p = 0.05	0.62	p < 0.05	2.05
Quinolinic acid	8.03	p = 0.05	0.87	p < 0.05	1.40

On D3 and D10 they were all found with no significant statistical alteration between the groups. ^#^Fold change was calculated based on the mean of each group (septic, control) by using the logarithm of the ratio septic/control for every significant metabolite at each time point.

**Table 3:** Metabolites found by LC-MS/MS to have significantly changed in septic neonates at the onset of the disease (D0) and their evolution in D3.

Metabolites	D0				D3			
	Confirmed		Possible		Confirmed		Possible	
	p value	log2fold change	p value	log2fold change	p value	log2fold change	p value	log2fold change
Valine	<10^−3^	4.01	<10^−2^	1.35	>0.05		<10^−2^	4.71
Phenylalanine	<10^−2^	2.91	<10^−2^	3.30	>0.05		<0.05	5.65
*Taurine	<0.05	1.27	<10^−2^	3.55	<10^−2^	2.75	>0.05	
g-aminobutyric acid	=0.05	1.09	<10^−2^	4.61	>0.05		>0.05	
Isoleucine	<10^−2^	2.74	<10^−2^	2.59	<10^−2^	7.06	<10^−2^	4.44
Glutamic acid	=0.05	1.92	<10^−2^	2.66	>0.05		>0.05	
Hypotaurine	=0.05	2.59	<0.05	2.53	>0.05		>0.05	
Trimethylamine-N-oxide	<0.05	−1.98	<0.05	−3.46	<0.05	−3.06	<0.05	−3.03
*Pyruvic acid	=0.05	1.98	=10^−3^	1.94				
Fumaric acid	<10^−2^	−2.09	=0.05	−1.10	<10^−3^	−3.41	>0.05	
Lactic acid	<0.05	2.56	<10^−2^	3.37	>0.05		<0.05	5.04
Hippuric acid	=0.05	−2.14	<0.05	−1.46	>0.05		>0.05	
*D- Glucose	<10^−2^	1.06	<0.05	3.58	>0.05		>0.05	
Nicotinamide	<10^−2^	−3.38	<10^−2^	−2.75	<10^−2^	−2.47	<0.05	−2.67
Riboflavine	=0.05	−0.79	<10^−2^	−1.67	<10^−2^	−5.57	>0.05	
Thiamine	<0.05	−1.61	=0.05	−0.72	<0.05	−1.28	>0.05	
*Inosine	<10^−2^	1.34	<10^−2^	2.74	>0.05		>0.05	

On D10 they were all found with no significant statistical alteration between the groups.

*Parallel significant change of the metabolite on ^1^H-NMR data.

Log2fold change was calculated based on the mean of each group (control, septic) by using the logarithm (base 2) of the ratio septic/control for every significant metabolite at each time point.
