# Supplementary material for: Urine metabolomics in neonates with late-onset sepsis in a case-control study
Source: Sci Rep. 2017 Apr 4;7:45506. doi: 10.1038/srep45506 (PMC5379623; doi:10.1038/srep45506)

URINE METABOLOMICS IN NEONATES WITH LATE-ONSET SEPSIS IN A CASE-CONTROL STUDY

Kosmas Sarafidis[1#,*], Anastasia Chrysovalantou Chatziioannou[2,#], Agathi Thomaidou[1], Helen Gika[3], Emmanouel Mikros[4], Dimitra Benaki[4], Elisavet Diamanti[1], Charalampos Agakidis[1], Nikolaos Raikos[3], Vasiliki Drossou[1], Georgios Theodoridis[2,*]

[1]1st Department of Neonatology, School of Medicine, Aristotle University of Thessaloniki, Greece
[2]School of Chemistry, Aristotle University of Thessaloniki, Greece
[3]Laboratory of Forensic Medicine and Toxicology, School of Medicine, Aristotle University of Thessaloniki, Greece,
[4]School of Pharmacy, National and Kapodistrian University of Athens, Greece

Corresponding authors:
*(Clinical data) Kosmas Sarafidis, M.D., Associate Professor in Neonatology, Aristotle University of Thessaloniki, Greece
Email: saraf@med.auth.gr
Tel: +30 2310992993
Fax: +30 2310992878

*(Metabolomics) Georgios Theodoridis, Professor in Analytical Chemistry, Aristotle University of Thessaloniki, Greece
Email: gtheodor@chem.auth.gr
Tel: +30 2310997718
Fax: +30 2310997719

# These authors contributed equally to this work

This file contains supplementary information on Materials and Methods:
Materials, Standard solutions, Sample preparation, HILIC-MS/MS analysis and 1H NMR analysis
The file also contains five figures as supplementary information:
Fig. S.1 ROC curves and box plots of significant metabolites found by ^{1}H-NMR
Fig. S.2 OPLS-DA plot of day 0 samples on LC-MS/MS on UV scaling. Green boxes have been used for controls and red circles for sepsis samples. Clear separation of two groups is observed according to the valid permutation plot (insert figure, n=999 permutations).
Fig. S.5 Loadings of
Fig. S.4 OPLS-DA plot of day 3 samples on LC-MS/MS on UV scaling. Green boxes have been used for controls and red circles for sepsis samples. Clear separation of two groups is still observed according to the valid permutation plot (insert figure, n=999 permutations).
Fig. S.5 ROC curves and box plots of significant metabolites found by LC-MS/MS

Materials

Deionized ultrapure water (18.2 MΩ cm^{-1}) produced by a Milli-Q Direct-Q 3 UV water production system (Millipore International, Merck KGaA, Darmstadt, Germany) was used to prepare all solution. Acetonitrile used for protein precipitation and analysis was of LC/MS analytical grade (Carlo Erba, Val de Remil, Frence). Formic acid, ammonium formate of LC/MS grade, deuterium oxide (D_2O, 99.9%), sodium azide (NaN_3) and 3-(Trimethylsilyl)propionic-2,2,3,3-d_4 acid (TSP, 98%) was purchased from Sigma Aldrich. All standards were of analytical or higher grade, and were purchased from various sources.

Standard solutions

Stock solutions of the 108 pre-selected metabolites were prepared based on the Sampsonidis et al. and Virgiliou et al. (J. Chrom. A 2015 and Electrophoresis 2015) protocol, in concentrations of 1000 mg/L in methanol/water, 1:1 (v/v) or water, depending on analyte solubility. In certain cases addition of minor amounts of base or acid, heating or sonication was needed to assist dissolution. All stock solutions were stored in glass vials at -24°C. Working standards were prepared from the stock solution by appropriate dilution with acetonitrile / water, 95:5 (v/v). Since metabolites exist in the real urine samples of interest in a wide concentration range they were classified in three different concentration groups based on their signal on LC-MS/MS: group A (concentrations 0.01-2 mg/L), group B (0.2-18 mg/L) and group C (5-95 mg/L).

As quality control (QC) sample, a standard mixture solution with analyte concentrations in the middle the concentration level of the calibration range, was used to study method inter/intra assay precision and accuracy.

Sample preparation

Upon collection samples were centrifuged (2500 g, 15 min) and supernatants were stored at -80° C till analysis.

Sample preparation of urine samples for HILIC-UHPLC-MS/MS analysis requires protein precipitation. Thus, 50 μL of each sample were diluted with 150 μL of acetonitrile and, after vortexing for 1 min, the mixture was centrifuged at 7000g for 10 min to remove precipitated proteins and particulate matter. Urine samples that were to be analyzed in a single instrument run were thawed just prior to processing. After the

protein precipitation, 100 μL of each clear supernatant was transferred to LC/MS vial, while 30 μL were mixed with equal volumes from the supernatants of each sample in a glass vial for the preparation of a biological quality control (QC) sample. Prior to the batch analysis injections of that QC were performed for column and system's conditioning purposes, while one QC samples was analyzed every ten injections as a control point of the system stability. Two other phenotypic QCs were prepared by mixing 30 μL of the supernatant of each group ($QC_{controls}$ and QC_{sepsis}). All samples, QCs and phenotypic QCs were treated similarly and were randomly loaded on the autosampler tray. The tray was thermostatically regulated at 6°C during analysis.

Sample preparation of urine samples for NMR analysis was performed by adapting the protocol of urine NMR metabolomics analysis from Bruker, due to limited volumes of samples available from neonates. After thawing, samples were centrifuged at 12000 g for 12 min. A supernatant of 400 μL of each centrifuged urine sample was mixed with 195 μL of double-distilled water and 65 μL of 1.5 mol/L phosphate urine buffer in D_2O, pH 7.4, containing 2 mmol/L NaN_3 and and 12.5 mg/mL TSP. The mixture was vortexed and centrifuged at 12000 g for 12 min. 550 μL of the supernatant was transferred into 5 mm NMR tubes. Also, an amount of 20 μL from each sample was used to form the QC sample of our analysis. All samples and QCs were treated similarly and were randomly loaded on the autosampler tray on the NMR, with the only restriction of a QC sample every 10 real samples.

Methods and instrumentation

HILIC-MS/MS analysis

The chromatographic separation was conducted on an Acquity UHPLC system (Waters Corporation, Millford, USA). The Acquity UHPLC system consists of an autosampler with a refrigerated storage space for vials operating at 6°C, an oven for the column temperature equalization. The Acquity BEH Amide Column (2.1 mm x 150 mm, 1.7 μm particle size) and the Acquity UPLC Van-Guard Pre-column (Waters Ltd, Elstree UK) were maintained at 40° C during the analysis. A volume of 5 μL of each sample was analysed under gradient elution of a binary solvent system. Solvent A (Acetonitrile:Water 95:5) and solvent B (Water: Acetonitrile 70:30) both contained 10 mM ammonium formate. The gradient elution profile

applied was starting with 100% A until 4 min, linearly changing to 60% A until 25 min, 15% A at 30 min, changing to the initial composition of 100% A at 30.01 min and constant till 40 min and the next injection. The optimum chromatographic separation was achieved with constant flow of 500 μL/min. The injection syringe and valves were washed before each circle with a weak solvent (Water:Methanol 70:30, 0.1% formate) and a strong solvent (Acetonitrile:Water 95:5, 0.1% formate) and after each injection with strong solvent.

Targeted MS-MS data were acquired on Xevo TQD system (Waters Corporation, Milford, USA) in polarity switching operation of the electrospray ionization for a 40 min analysis. Capillary voltage was set at +3500 V or -3500 V for each ionization mode and the polarity switching time was automatically adjusted. Compound dependent parameters were optimized for each of the 108 studied metabolites separately in direct infusion mode. Block and Desolvation temperatures was set at 350° C and desolvation gas flow rate at 650 L/h. Block temperature was 150° C and cone gas flow was set at 50 L/h. Multiple reaction monitoring (MRM) mode was applied for the detection and quantification of all the compounds.

The raw spectrometric data analysis was performed with Waters MassLynx and TargetLynx software ver. 4.1. The integration of the proper peak for each compound was ensured by the comparison of the retention time of each analyte with a spiked standard to urine QC matrix. TargetLynx settings were optimized for each compound to achieve the optimum automated integration for every target.

Results were exported as .txt files from TargetLynx. Due to the incompatible format of that exports with the multivariate statistical software, excel visual basic scripts were developed to accelerate and ensure the accuracy of the final data table. The resulted data tables were subjected to univariate statistical analysis by t-test and multivariate analysis by SIMCA 13 (Umetrics, Umea, Sweden). In the present study the significance level was chosen to be 0.05. Thus, as significant metabolites were considered only those compounds having a t-test p-value lower than 0.05.

Datasets were analyzed in Simca 13 using UV scaling. UV scaling was finally utilized because the resulted models where of better quality compared to e.g. Pareto scaling. Furthermore, UV is the most commonly used scaling tool for targeted MS metabolomics studies QC samples were tightly grouped together in the center of the PCA scores plot, showing a reasonably good stability of the chromatographic system.

^{1}H NMR analysis

All NMR experiments were recorded on a Bruker Avance III 600 MHz spectrometer, employing an inverse detection probe (5 mm) with z-gradients. One-dimensional ^{1}H NMR spectra were obtained using a standard 1D NOESY pulse sequence (noesypr1d) for water suppression. For each samples 128 scans were collected in 64-k data points over a spectral width of 14 ppm. All spectra were referenced to the chemical shift of TSP at δ 0.0 ppm and baseline-corrected by applying a polynomial curve fitting using the XWIN-NMR software (Bruker BioSpin GmbH). Univariate and multivariate statistics were again applied in order to reveal the differences between the selected integrated peaks.

Fig. S.1 ROC curves and box plots of significant metabolites found by ¹H-NMR

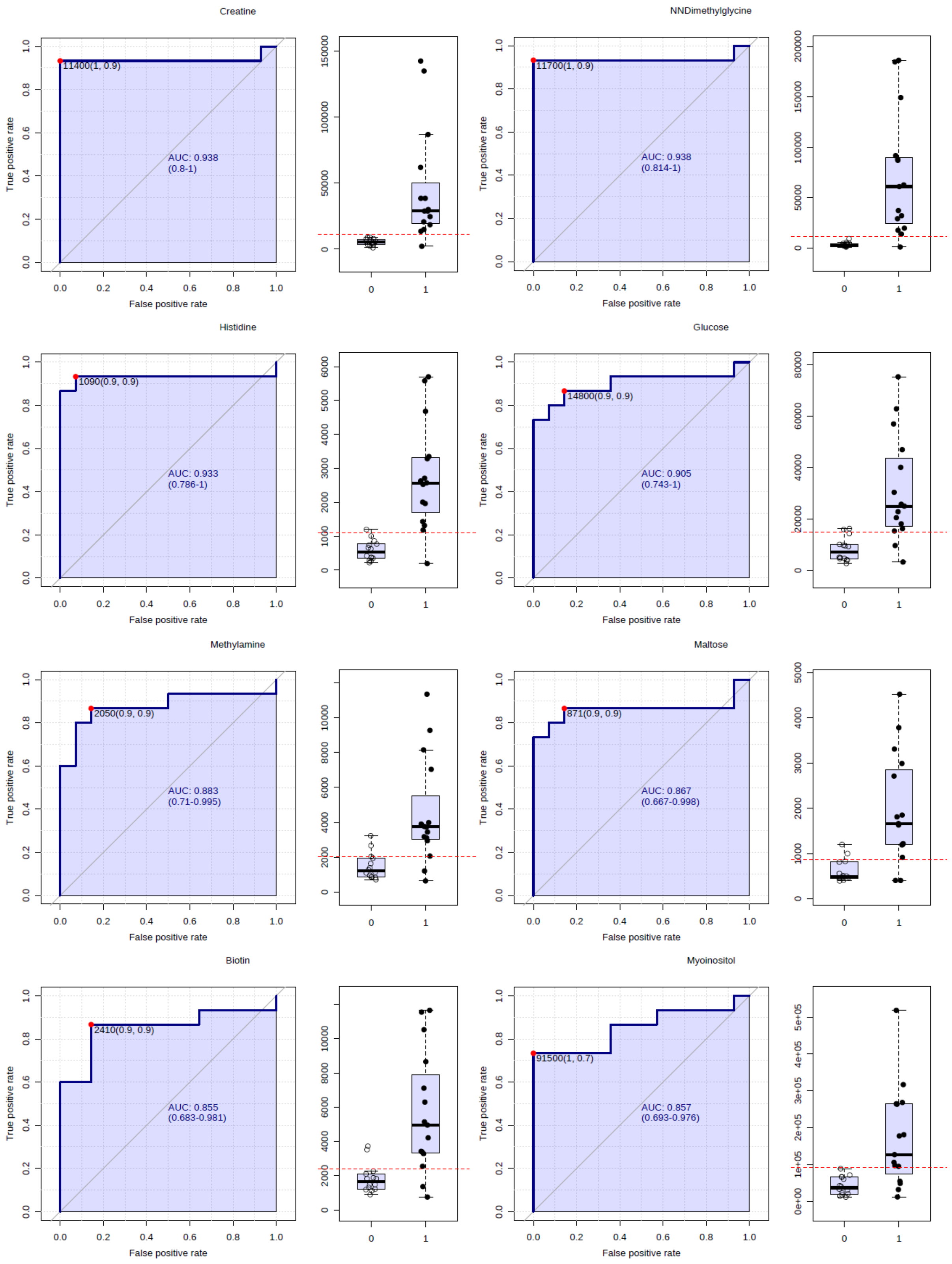

Fig. S.2 OPLS-DA plot of day 0 samples on LC-MS/MS on UV scaling. Green boxes have been used for controls and red circles for sepsis samples. Clear separation of two groups is observed according to the valid permutation plot (insert figure, n=999 permutations).

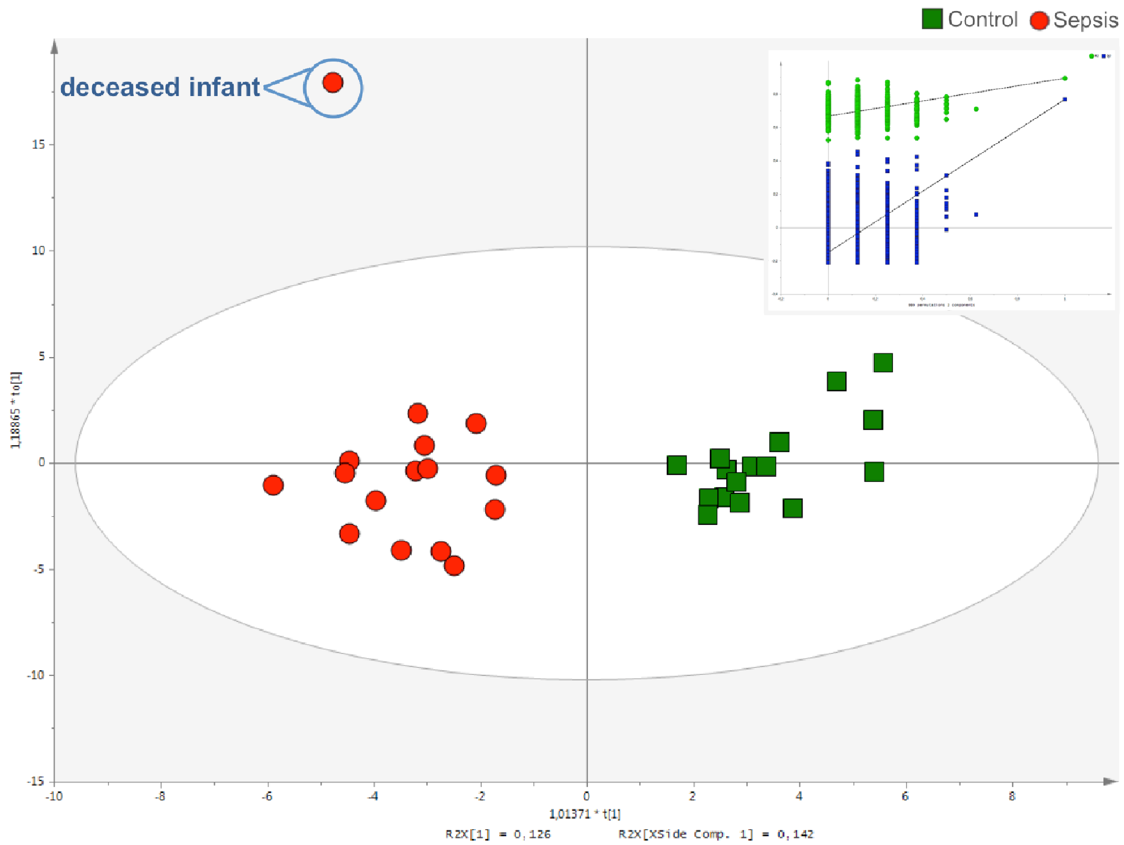

Fig. S.3 OPLS-DA loadings plot of day 0 samples generated by LC-MS/MS on UV scaling. Metabolites depicted on the edge of the plot contribute to the differentiation of samples: metabolites on the left show higher signals on sepsis cases, while metabolites on the right part of the plot are higher in controls. These metabolites were also highlighted in the application of all evaluation criteria applied (t-test, log2foldchange, VIP, box plots).

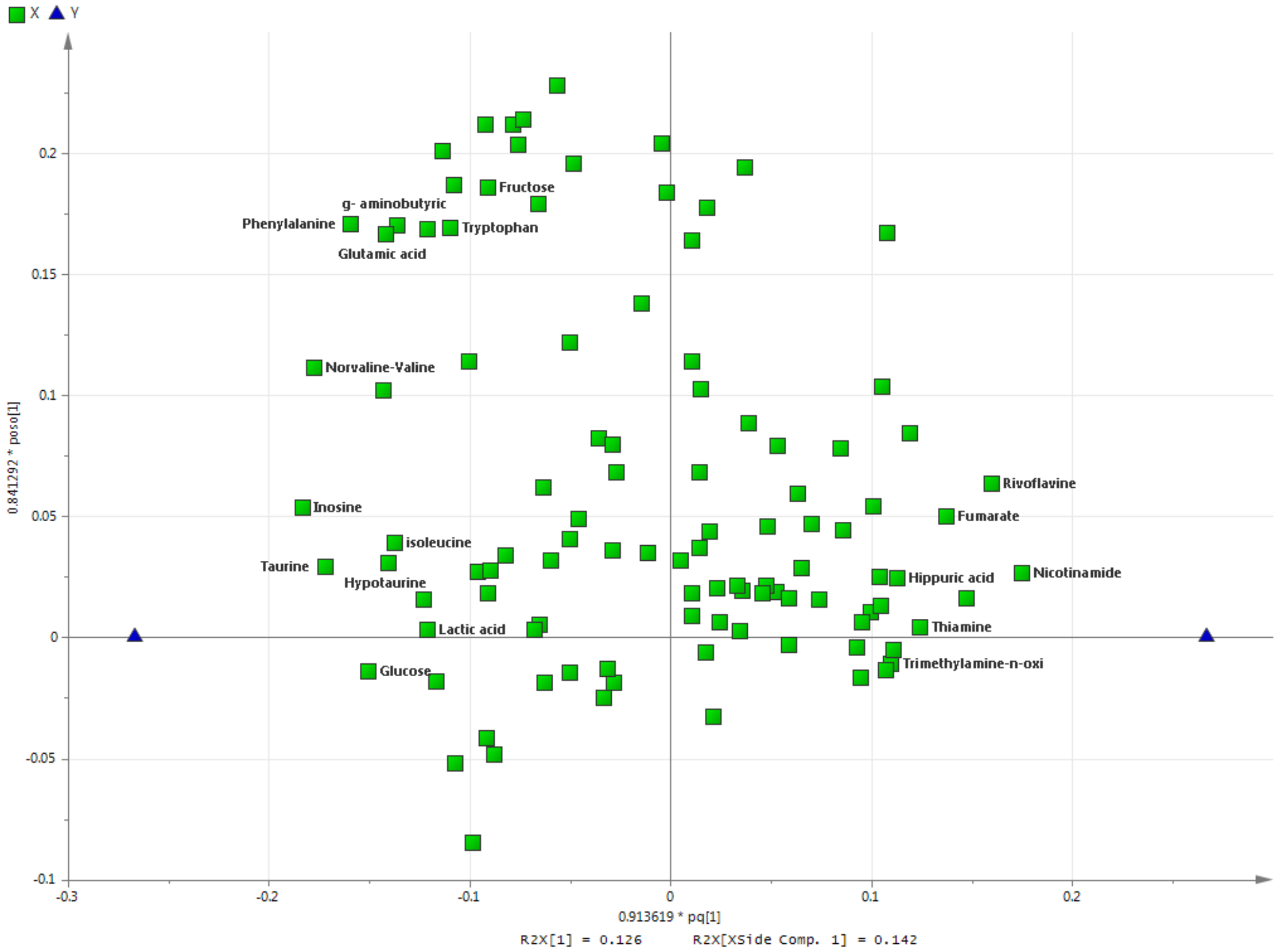

Fig. S.4 OPLS-DA plot of day 3 samples on LC-MS/MS on UV scaling. Green boxes have been used for controls and red circles for sepsis samples. Clear separation of two groups is still observed according to the valid permutation plot (insert figure, n=999 permutations).

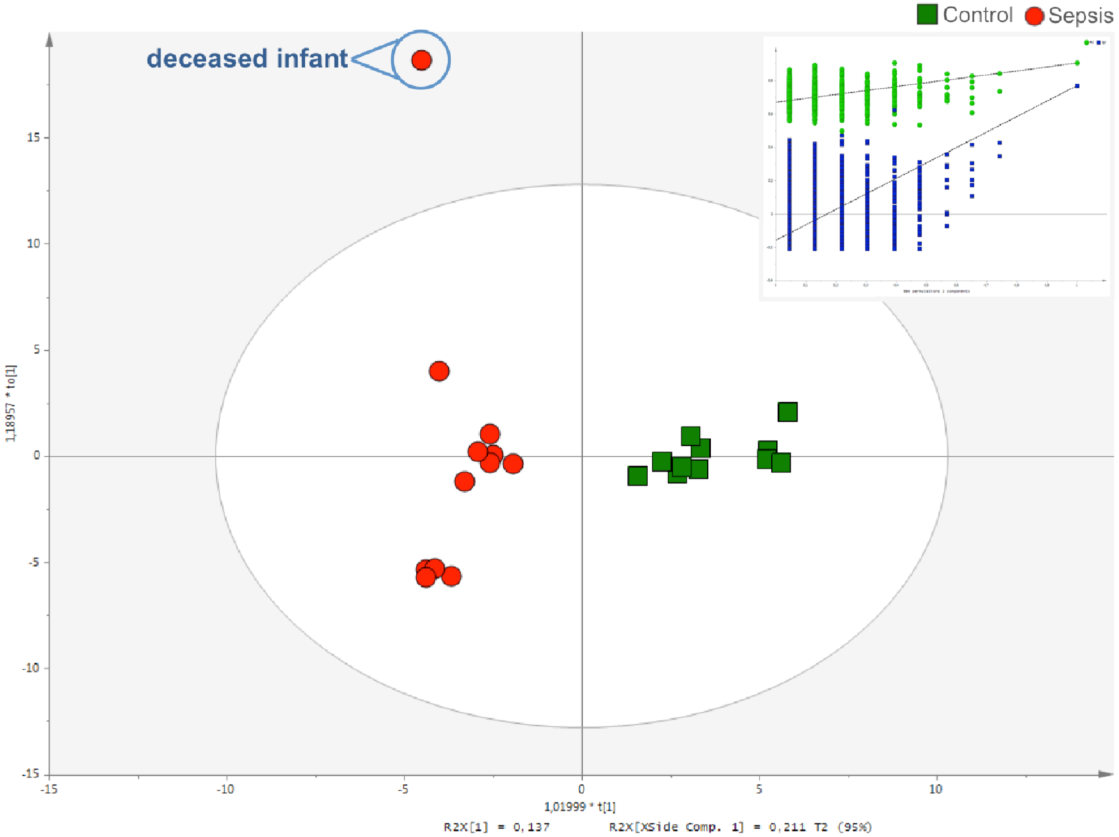

Fig. S.5 ROC curves and box plots of significant metabolites found by LC-MS/MS

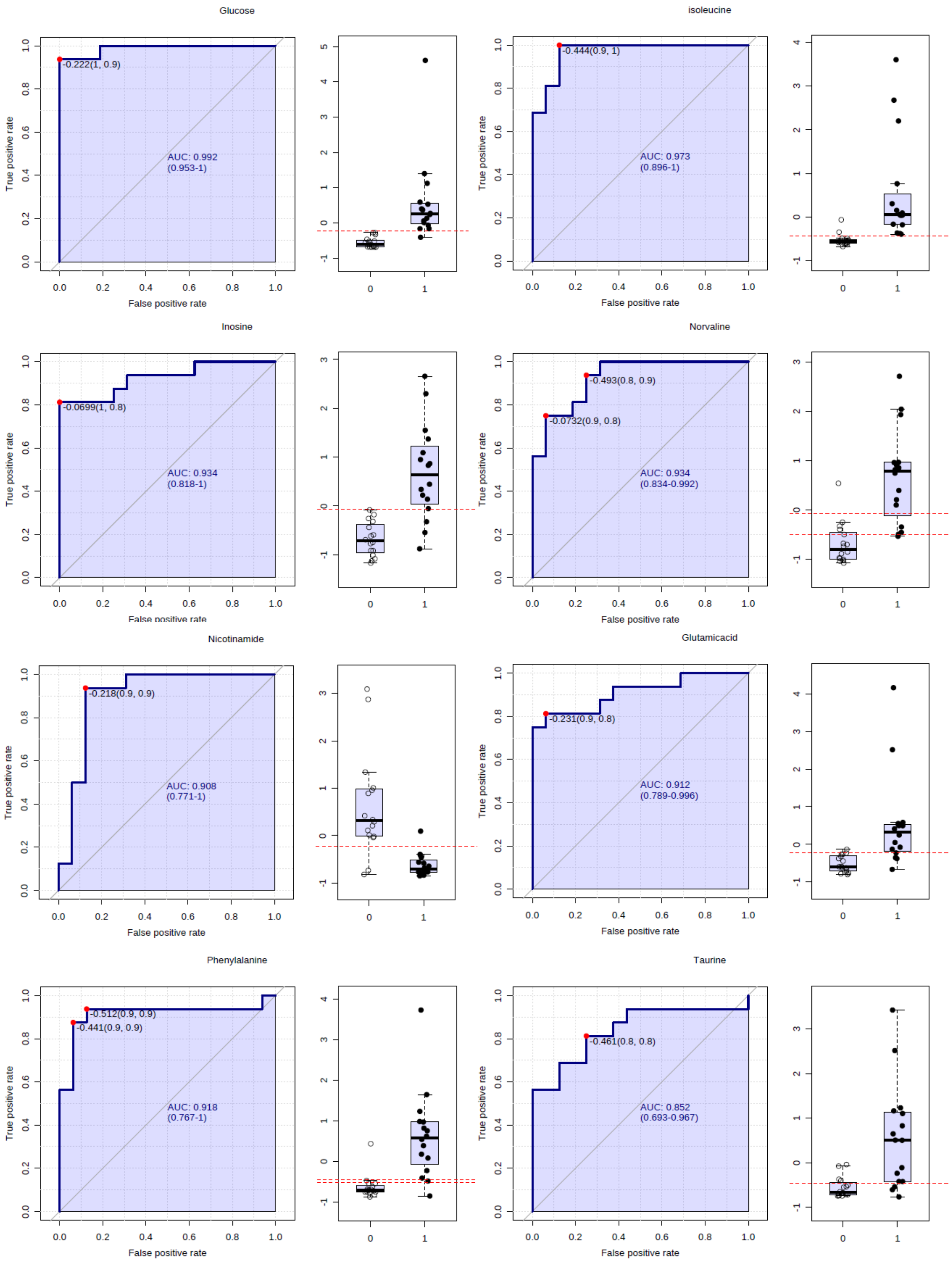

Supplement: Supplementary Information [file srep45506-s1.pdf]
